# The impact of invisible-spreaders on COVID-19 transmission and work resumption

**DOI:** 10.1371/journal.pone.0252994

**Published:** 2022-01-12

**Authors:** Chao Wu, Cong Xu, Feng Mao, Xiaolin Xu, Chan Zhang

**Affiliations:** 1 School of Public Affairs, Zhejiang University, Hangzhou, China; 2 Data Science Institute, Imperial College London, London, United Kingdom; 3 School of Earth and Environmental Sciences Cardiff University, Cardiff, United Kingdom; 4 Center for Biostatistics, Bioinformatics, and Big Data, Second Affiliated Hospital and Department of Big Data in Health Science, School of Public Health, Zhejiang University School of Medicine, Hangzhou, Zhejiang, China; 5 National Institute for Data Science in Health and Medicine, Hangzhou, Zhejiang, China; 6 College of Media and International Culture, Zhejiang University, Hangzhou, China; Frankfurt Institute for Advanced Studies, GERMANY

## Abstract

The global impact of coronavirus disease 2019 (COVID-19) is unprecedented, and many control and prevention measures have been implemented to test for and trace COVID-19. However, invisible-spreaders, who are associated with nucleic acid detection and asymptomatic infections, have received insufficient attention in the current COVID-19 control efforts. In this paper, we analyze the time series infection data for Italy, Germany, Brazil, India and Sweden since the first wave outbreak to address the following issues through a series of experiments. We conclude that: 1) As of June 1, 2020, the proportion of invisible-spreaders is close to 0.4% in Sweden, 0.8% in early Italy and Germany, and 0.4% in the middle and late stages. However, in Brazil and India, the proportion still shows a gradual upward trend; 2) During the spread of this pandemic, even a slight increase in the proportion of invisible-spreaders could have large implications for the health of the community; and 3) On resuming work, the pandemic intervention measures will be relaxed, and invisible-spreaders will cause a new round of outbreaks.

## I. Introduction

The global impact of coronavirus disease 2019 (COVID- 19) has been unprecedented. As of Jun 1, 2020, an outbreak of COVID-19 has resulted in 6,016,976 confirmed cases and 370,153 deaths [1, 2]. In order to control the pandemic, many measures have been taken, including: *The Closure of workplaces*, *schools and universities; The social distancing of entire populations; Voluntary home quarantine; and Lockdown* [3]. However, these measures cannot eliminate the problem of false-negative individuals.

As the peak of the pandemic has passed in some countries, an imminent trend to resume work is expected. Therefore, the above control measures in these countries may consequently be relaxed. However, there is a serious problem that people may not realize: there are false-negative individuals in the population, whose impact on COVID-19 remains to be studied. The false-negatives mentioned in this article are not caused solely by incorrect test results in the traditional sense. We will define false-negative individuals in a broader sense as *invisible-spreaders*: when a person is infected with COVID-19, but for some reason cannot be effectively detected and isolated, and always remains infectious toward other people, he/she will be defined as an *invisible-spreader*.

According to the literature, there are two main sources of *invisible-spreaders* [4, 5]:

1. Nucleic acid detection: According to the recommendations of the World Health Organization (WHO), the main diagnostic method for COVID-19 is nucleic acid detection in fluid secretion collected via a pharyngeal swab using RT–PCR [6]. However, nucleic acid detection based on pharyngeal swabs has an accuracy rate of 71% [7]. Several factors might have contributed to these false-negative results, such as the sampling technique, transportation process, or limited gene(s) detection [8].
2. Asymptomatic: Evidence has emerged from certain countries like Italy that indicates that some coronavirus infections do not result in symptoms [9]. These asymptomatic infections cannot be recognized without nucleic acid detection. Before nucleic acid detection, these asymptomatic infections have been infectious to susceptible people. However, it is unrealistic to carry out nucleic acid detection for the whole population, so some people with asymptomatic infection become super-spreaders.

Undoubtedly, these *invisible-spreaders* will have a direct implication regarding the control of the pandemic, and may even lead to the resurgence of the pandemic [10]. In this article, we explore the following issues:

*Proportion*: The impacts of different proportions of *invisible-spreaders* on the pandemic differ. Therefore, to evaluate more accurately the development of the pandemic, we estimated the proportion of *invisible-spreaders* in various countries through machine learning.

*The impact of invisible-spreaders*: During COVID-19, those with confirmed infection were quarantined. However, *invisible-spreaders* cannot be quarantined in time due to the lack of effective means. Therefore, they would infect their close contacts while infectious, which accelerates the spread of the virus. In order to explore the effects of *invisible-spreaders*, we designed a series of controlled experiments. By changing the parameters of the proportion of *invisible-spreaders*, several pandemic simulation experiments were performed to explore the various effects of different proportions of *invisible-spreaders* on the pandemic.

*New outbreak*: The peak of the pandemic has passed in some countries, like Italy and Germany. Italy relaxed its outbreak control measures twice (on April 14 and May 4, 2020) [11], and Germany reopened shops and Bundesliga football as the lockdown was relaxed on May 6, 2020 [12]. Nevertheless, due to the existence of *invisible-spreaders*, it is uncertain how the pandemic will develop. Therefore, we have simulated the development of COVID-19 in these countries following the relaxation of the control measures to provide a reference for these countries to formulate reasonable plans.

We summarize our contributes below:

- We tried to conduct a quantitative analysis of COVID-19 and use data to illustrate the various effects of different proportions of *invisible-spreaders* on the pandemic.
- We define a modified SIR model to simulate the spread of COVID-19 among five different countries. More in detail, we use model parameters that change over time and include *invisible-spreader* as a new variable in our model.
- We propose a simple method to estimate the proportion of *invisible-spreaders*.
- Based on the existing pandemic data, we used experiments to predict the development of the pandemic after the intervention measures were relaxed.

The rest of this work is structured as follows. In Section II, we first introduce the SIR model [13, 14], which describes its formulas and concepts; then, we will focus on explaining our model. In Section III, we presented the results of each experiment and appropriately analyzed them. In Section IV, we discussed more sources of false negatives and future research directions. In Section V, we concluded the article.

## II. Methods

### A. Data sources

The first wave global COVID-19 data were collected from the Johns Hopkins University’s Report [15]. Five countries (Italy, Germany, Brazil, India, and Sweden) were chosen to represent a variety of different pandemic situations. Italy, Germany, and Sweden were the three more severely affected countries in the first wave of pandemic in Europe, and the control measure adopted by Sweden was group immunization [16]. Brazil and India were chosen as countries where the epidemic is still on the rise. And all data was completely anonymized and de-identified before access and analysis.

To estimate the impact and proportion of *invisible-spreaders*, we considered time series information of the first wave pandemic in each country from January 22 to May 31, 2020. However, in the early stage of the pandemic, the medical institutions had not yet developed a complete system for reporting cases, which will lead to data deviation. In addition, after a period of outbreaks, countries undertake interventions against COVID-19 which will make the daily infection factor show a downward trend. During this period, the impact of *invisible-spreaders* will be more prominent because, following interventions, positive patients will be detected and quarantined, but *invisible-spreaders* can still be an effective source of infection. Therefore, we selected data for a reasonable time period (Table 1). Since there were only 24 new patients in India on April 3, which is close to a twentieth of the increase on other days in the previous week, we believe that there are some deviations in the statistics for that day, so we used Lagrange interpolation to complete the data for the day [17].

**Table 1:** Range of data for five countries: Italy, Germany, Brazil, India and Sweden.

Country	Start	End
**Italy**	February 23, 2020	June 1, 2020
**Germany**	March 1, 2020	June 1, 2020
**Brazil**	March 19, 2020	June 1, 2020
**India**	March 5, 2020	June 1, 2020
**Sweden**	March 7, 2020	June 1, 2020

### B. Models

Many mathematical models have been applied to the study of COVID-19, such as the SIR model [13, 14], and the SEIR model [13]. However, these models all adopt fixed parameters without considering the interventions and time decay during the pandemic progression. For this reason, we adopt a modified SIR model.

1) *Standard SIR Model*: In this model, the pandemic transmission is treated as a geometric random walk process. This means that, at the same time, the probability of infection is considered constant in this model. The standard SIR epidemiological model divides individuals into three classes, as follows: susceptible (S), infected (I) and removed (R).

dStdt=-βNStIt,

R0=β∙DI,

dItdt=βNStIt-γIt,

dRtdt=γIt (1)

Where *S*(*t*), *I*(*t*) and *R*(*t*) are the number of susceptible, infections and removed individuals at time t; N is the population size, *N* = *S*(*t*) + *I*(*t*) + *R*(*t*); *R*_0_ is the basic reproduction number [18]; *D*_*I*_ is the mean infection period; *β* is the infection factor, *γ* is the removed factor.

The infection factor *β* (The product of the contact rate and the infection rate) and removed factor *γ* are constant in the SIR basic model. However, these factors are not static in real life. They change along with the development of the pandemic, and are dependent on the pandemic prevention strategies adopted by the government. For example, the infection factor can be decreased if people are requested to maintain a safe distance from others in order to slow the spread. Moreover, the basic model does not consider *invisible-spreaders*, which harms the control of the pandemic.

2) *Modified SIR Model*: The modified model divides the population into the following four classes: susceptible (S), confirmed (C), *invisible-spreaders* (F) and removed (R). In order to adapt the model to real-life situation, we use an infection factor that changes over time. The infection factor is assumed to be a random variable, described by a function *β*(*t*).

βt=atb+c (2)

a, b and c are the bias terms of the model relative to the time dimension, referring to the bias terms in the neural network to prevent the model from failing to converge.

The data we obtain is based on daily reports and, once the cases are reported, this means that they have been quarantined. Isolated cases will lose their infectivity to susceptible people, just like recovered people. Therefore, we don’t need to consider the recovery rate γ, and the process of daily case addition will be regarded as Markov process [19]. We add a new *invisible-spreader* factor to indicate the probability that the infected patient is an *invisible-spreader*, and assume that the recovery time of an *invisible-spreaders* is 14 days.

dStdt=-βtPStCt+Ft, (3)

R0=βt∙DI, (4)

dCtdt=βtPStCt+Ft1-α, (5)

dFtdt=αβ(t)PStCt+Ft (6)

Where *S*(*t*), *I*(*t*), *F*(*t*) and *R*(*t*) are the number of susceptible, infected, *invisible-spreaders* and removed individuals at time t; P is the number of the existing population in the system, *P* = *S*(*t*) + *I*(*t*) + *F*(*t*); *α* is the *invisible-spreader* rate; Since the infection process is a Markov process, *D*_*I*_ equals to 1, *R*_0_ equals to *β*(*t*).

We first use the data for Italy, Germany, Brazil, India, and Sweden according Table 1 to train our model and use the stochastic gradient descent (SGD) to calculate the appropriate parameters [20]. Then, four groups of control experiments were carried out using the trained model. In the first group of experiments, we used the number of new infections of real data as ground truth, and machine learning to fit ground truth, then the loss function L is:

L=12n∑t|C-t-Ct|2 (7)

where $\overset{-}{C}(t)$ is the real data of new infections, n is the number of training data, t is the duration of the pandemic.

## III. Result

In order to calculate the proportion of *invisible-spreaders* and verify the influence of *invisible-spreaders*, we conducted multiple experiments. Through SGD, we can approximately calculate the proportion of invisible-spreaders in real-life situations. Secondly, since the proportion of early invisible-spreaders in Germany and Italy is close to 0.8%, and the proportion in Italy, Germany and Sweden, after the peak of the pandemic has passed, is close to 0.4%, simulation experiments were carried out under the assumption that the proportion (α) is equal to 0%, 0.4%, and 0.8%. Under these three proportions, we simulated the pandemic’s progression in five countries, and evaluated the impact of invisible-spreaders through the differences among the three proportions. Thirdly, with the improved situation in some countries regarding the pandemic, they gradually relaxed the pandemic interventions. Due to the existence of invisible-spreaders, there is a risk of a new pandemic outbreak if these interventions are relaxed. Therefore, to evaluate the damage of the new outbreak, we used data for June 1 as the first day data to initialize the model, and conducted a simulation experiment of the pandemic.

### A. Infection factor

**Fig 1:**
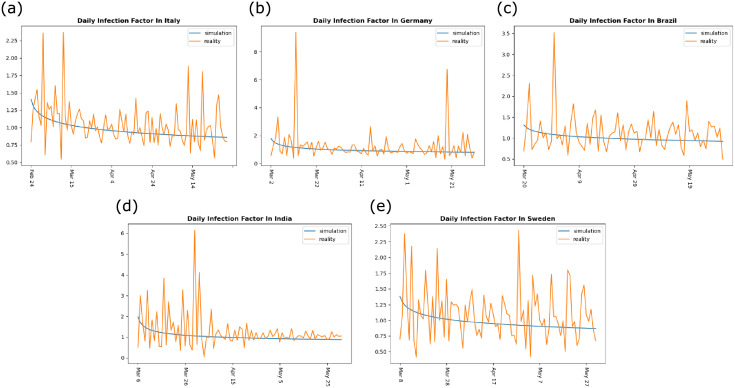
Daily infection factor. (a) daily infection factor change in Italy since February 23, 2020, (b) daily infection factor change in Germany since March 1, 2020, (c) daily infection factor change in Brazil since March 19, 2020, (d) daily infection factor change in India since March 5, 2020, and (e) daily infection factor change in Sweden since March 7, 2020. The orange curve describes the infection factor in the real world, and the blue curve is the simulated infection factor obtained through regression analysis.

The model simulated the pandemic’s progression in Italy, Germany, Brazil, India, and Sweden over time. Then, by establishing a regression model, taking the number of daily infections as ground truth, and fitting the predicted value of the model to the ground truth, an approximate curve for the infection factor was obtained (Fig 1). Along with the development of the pandemic, Italy, Germany, Brazil, India, and Sweden have gradually adopted several interventions. Therefore, the infection factors should be inversely related to time. As the figure shows, although the infection factors in these five countries follow a downward trend, there are always fluctuations in the curves. After entering May, moreover, the outbreak in Italy, Germany, and Sweden shows a rebound trend. This is because Italy and Germany both relaxed their pandemic intervention measures in May, and the herd immunity measures adopted by Sweden cannot effectively control the impact of *invisible-spreaders*.

### B. Proportion

Our experimental results show that the proportion of *invisible-spreaders* was less than 1% in these five countries. Among these five countries, although Sweden has the lowest proportion of *invisible-spreaders*, its curve is the most volatile. The reason is that herd immunity does not cut off the spread of *invisible-spreaders*. The situation in Italy and Germany is somewhat similar, with 0.8% in the early stage and stable to 0.4% in the middle and late stages. India and Brazil are experiencing the early conditions in Italy and Germany, and the proportion is still rising. This may occur since Brazil’s total population is far larger than that of Italy and Germany, and they are still in the spreading period of the pandemic.

### C. Impact of invisible-spreaders

**Fig 2:**
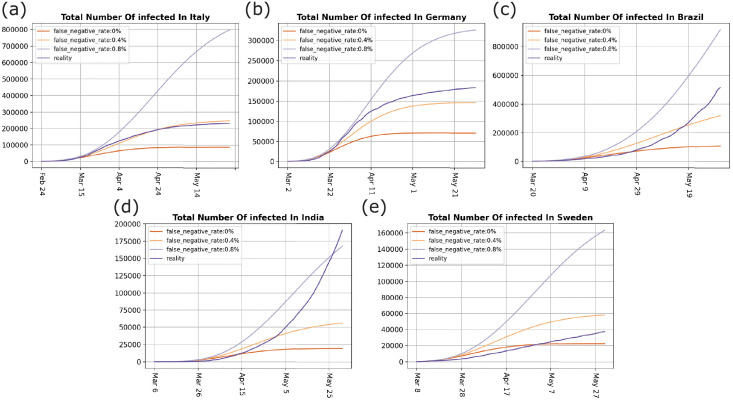
Total number of infected individuals. (a) total number of infected in Italy, (b) total number of infected in Germany, (c) total number of infected in Brazil, (d) total number of infected in India, and (e) total number of infected in Sweden. The red curve describes the total number of infected in the real world, and the other curves are the simulated total number of infected.

To estimate the impact of *invisible-spreaders*, we assumed the presence of 0%, 0.4%, and 0.8% *invisible-spreaders* among infected patients, and conducted five simulation experiments. The experimental results show that even a slight increase in the proportion of *invisible-spreaders* could have large implications for the health of the community (Table 2). If the proportion of *invisible-spreaders* increases to 0.8% from 0.4%, the total number of infections as of June 1 will increase to doubles or triples, and will also lead to a longer duration of the pandemic. This result is due to the fact that *invisible-spreaders* remained active in the model system until recovery. In the meantime, susceptible populations will continue to be infected by these *invisible-spreaders*, resulting in a higher number of daily infections compared with populations without *invisible-spreaders* (Fig 2). Our results suggest that *invisible-spreaders* enhance the spread of the pandemic, even in the context of prevention and control measures.

**Table 2:** Total number of infections in Italy, Germany, Brazil, India, and Sweden under 0%, 0.4%, and 0.8% *invisible-spreader* proportions up to June 1, 2020.

Country	Proportion of *invisible-spreaders*	Total infections
**Italy**	0%	87428
**Italy**	0.4%	247062
**Italy**	0.8%	796811
**Germany**	0%	70989
**Germany**	0.4%	146259
**Germany**	0.8%	326013
**Brazil**	0%	108947
**Brazil**	0.4%	320136
**Brazil**	0.8%	914325
**India**	0%	19433
**India**	0.4%	56001
**India**	0.8%	167505
**Sweden**	0%	22573
**Sweden**	0.4%	58090
**Sweden**	0.8%	163214

**Fig 3:**
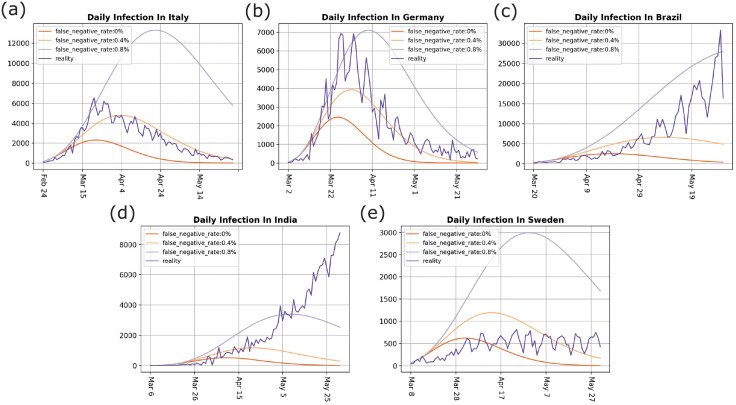
Daily infections. (a) daily infections in Italy, (b) daily infections in Germany, (c) daily infections in Brazil, (d) daily infections in India, and (e) daily infections in Sweden. The red curve describes the daily infections in the real world, and the other curves are the simulated daily infections.

Figs 2 and 3 show that the proportion of *invisible-spreaders* in Germany is slightly higher than in Italy, and the population of Germany is also denser than that of Italy. Therefore, we might assume that the pandemic would be more severe in Germany, yet the reality is the opposite. The number of infections is far greater in Italy, and the inflection point also appears later. One of the reasons for this difference is that the intervention measures adopted by the two countries are different. On March 22, Germany began to implement interventions, including *banning parties*, *closing restaurants*, and imposing a *stay-at-home order*, which significantly reduced the contact between people and cut off transmission by *invisible-spreaders*. Italy only implemented t*he banning of large gatherings* and *the closure of schools*, so local community transmission and family cluster transmission may be increased when people have nowhere to go [21], which led to the pandemic being more affected by *invisible-spreaders* in Italy. The difference between these two countries indicates that a stay-at-home order enhances pandemic control. The pandemic in China and Germany was significantly controlled through the adoption of this measure. According to the current data (Table 1), the number of daily infections in Brazil and India is still increasing, so these two countries might learn from the pandemic interventions (like the stay-at-home order) implemented by countries like Germany. For Sweden, the proportion of *invisible-spreaders* is almost below 0.4% (Fig 3), and its population density is only about one-tenth of Germany’s and one-eighth of Italy’s. However, its final infection rate was close to 0.37%, almost twice that of Germany’s 0.22%, and close to Italy’s 0.38%. This is because Sweden adopted a group immunization approach. According to the experimental results, group immunization has not achieved an ideal pandemic control effect.

### D. New outbreak

**Fig 4:**
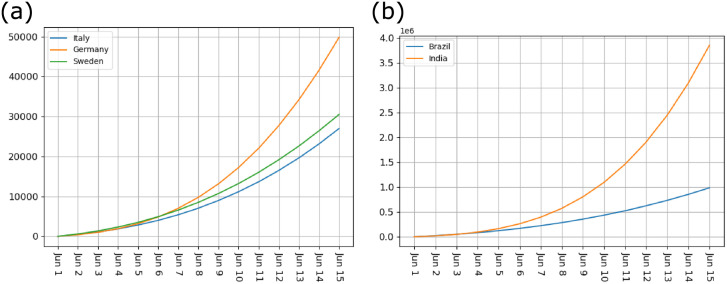
New outbreak. (a) new outbreak in Italy, Germany and Sweden, (b) new outbreak in India and Brazil.

According to the curves for the infection factor (Fig 1), after the beginning of May 2020, the pandemic tended to rebound. To examine the potential for new outbreaks, we used our model to simulate new outbreaks with 0.4% *invisible-spreaders*. As the pandemic interventions are relaxed, the daily infection factor will return to the mid-pandemic level. Therefore, We use June 1, 2020 as the first day to initialize the model parameters. We ran a simulation from June 1st to June 15th, and these five countries eventually reached a large number of new infections (Table 3). This result shows that, if the pandemic interventions are relaxed, then even if the proportion of *invisible-spreaders* is only 0.4%, the impact of the new outbreak will be catastrophic (Fig 4).

**Table 3:** Total number of new infections 14 days after the outbreak in Italy, Germany, Brazil, India, and Sweden, when the proportion of *invisible-spreaders* is 0.4%.

Country	New Infections
**Italy**	27021
**Germany**	49876
**Brazil**	983336
**India**	3846115
**Sweden**	30564

## IV. Discussion

In addition to the two main sources mentioned in this article, there are several other sources of *invisible-spreaders*. In order to handle this pandemic more effectively, China has developed a health code application, which is one of the ways to generate *invisible-spreaders*. In China, people can apply for a health code online using Alipay. After providing health-related information and confirming whether there has been any contact with a confirmed or suspected patient within the past fortnight, a color code is generated, after passing the audit. A person who receives a green code is safe and does not need to go into quarantine while a person who receives a red or yellow code should be quarantined according to the regulations until the health code changes to green. However, this self-reported information may not accurately reflect people’s health conditions, which means that some positive patients in the population will receive a green code, implying that some people who should have been isolated can travel freely. As far as the current situation in China is concerned, the health code is of great help in controlling the pandemic, and *invisible-spreaders* do not seem to have a negative impact. However, once China implements lockdown and only allows people with green codes to travel between cities, there will remain imported patients in various provinces and cities. Based on the experimental results discussed in this article, we believe that the reason why the health code does not appear to be having a negative effect in China is that China’s pandemic situation is developing positively. Once the peak of the pandemic in Italy, Germany, Brazil, India, and Sweden has passed, these countries also need to find a method similar to the health code to provide a logical basis for permitting travel as people resume work. If the health code is applied in these countries, where the pandemic situation is serious, it will exert a highly negative impact.

In addition to studying the issues outlined in this article, some studies believe that the infection power of asymptomatic cases is lower than that of symptomatic cases [3]. Therefore, it is necessary to classify *invisible-spreaders*. We plan to consider how to divide *invisible-spreaders* into three categories in future research: 1) false-negative for the nucleic acid test, 2) asymptomatic infection, 3) false-negative for the health code. Based on this classification, more reasonable interventions will be proposed.

There remains further research to be done. When modeling data from one country, the results show that the impact of different proportions of *invisible-spreaders* differs greatly and that a difference of only 0.4% will double the damage of the pandemic. The comparison of the data for Italy and Germany reveals a big gap between the impact of the same proportion of *invisible-spreaders*. Even if the proportion in Germany is greater than in Italy, Italy will suffer more losses than Germany. This is because the early interventions adopted by these two countries differ—the stay-at-home-order caused this difference. In Sweden, the proportion of *invisible-spreaders* is the lowest among the five countries, and its population is far less dense than that of the other four countries, but its final infection rate is close to that of Italy. This means that the effect of group immunization on COVID19 is not ideal. Moreover, the United Kingdom, which also proposed group immunization in the early stage, has also abandoned this measure. The above results show that to control the pandemic, it is vital to select and implement reasonable intervention measures. Therefore, further quantitative research on various interventions is required.

Many countries, such as Italy, Germany, Brazil, India, and Sweden, have adopted various interventions to reduce the damage caused by the pandemic. Moreover, the number of daily infections in Italy and Germany has gradually decreased from March 27 and April 3, respectively, which means that, in these two countries, the pandemic has been controlled to some extent. Under such circumstances, the resumption plan will enter the agenda of these two countries. However, there remain *invisible-spreaders* in the population, which will impede this plan. Unless we take measures to deal with false- negative individuals, they will lead to a new outbreak of the pandemic. Therefore, in order to reduce the negative impact of *invisible-spreaders*, we consider that the corresponding measures should be taken to combat the following three aspects:

1. Nucleic acid detection: The development and communication of clear, risk-stratified protocols to manage negative COVID-19 test results are needed [4]. Individuals should be divided into low-risk individuals and higher-risk individuals, based on whether they are from a hardest-hit area or have had close contact with an infected person within the previous fortnight. For low-risk individuals, a negative result for nucleic acid detection and green health code will be sufficient but, for higher-risk individuals, these results will be insufficient. After obtaining a negative result, these individuals may still need to quarantine and take further tests. A CT chest scan——A computerized tomography scan uses computers and rotating X-ray machines to create cross-sectional images of the body——and clinical symptoms should also be used to assist the detection of false- negative individuals.
2. Asymptomatic: It is difficult to implement nationwide nucleic acid detection. However, extensive nucleic acid detection in high-risk areas, like Wuhan, Codogno and North Rhine-Westphalia, is still necessary. From May 14, 2020, Wuhan initiated centralized nucleic acid detection citywide, which detected a total of 300 infected yet asymptomatic people [22]. This nucleic acid detection can dispel people’s concerns, which will provide an excellent foundation for the resumption of work in Wuhan. It also provides a feasible scheme for other countries to ensure the further combatting of the pandemic by applying nucleic acid detection in the hardest-hit areas.
3. Health code: In order to solve the problem of false- negatives caused by the health code, we refer to “The Privacy-Preserving Contact Tracing” launched by Google and Apple [23]. This enables the health code app periodically to communicate with other health code apps around it. In this way, it is possible to record each person’s close contacts within a certain period, which may provide an important basis for the classification of the health code.

## V. Conclusions

During the past few months, the COVID-19 pandemic has swept the world. We hope to promote research on the pandemic by proposing a quantitative analysis method. It can be seen from Fig 1 that the daily infection factor is showing a downward trend as a whole, but there is always a fluctuation in the curve. We believe that *invisible-spreader* are one of the reasons for this fluctuation. And our second experimental results show that the proportion of *invisible-spreaders* in the five countries mentioned in this article does not exceed 1%, and the degree of influence caused by *invisible-spreaders* varies in different countries. This reminds these countries that appropriate intervention measures should be taken to control the pandemic. In addition, the third experiment shows that there are indeed some *invisible-spreaders* in the current population, and only a small number of *invisible-spreaders* will result in a large number of cases in the community. And it will be difficult to end the pandemic unless effective measures can be taken to detect *invisible-spreaders*. When entering the stage of work resumption, pandemic intervention measures will be gradually relaxed. During this period, the impact of *invisible-spreaders* will be more significant, and it is likely to cause a new outbreak. Therefore, these countries need to take corresponding measures to ensure the normal progress of work resumption.
